# Clinical Features and Histopathological Analysis of Oral Lichen Planus: An Analysis of 105 Chinese Patients

**DOI:** 10.3290/j.ohpd.b5570957

**Published:** 2024-07-12

**Authors:** Qin Liu, Hong Liu, Yifan Zhou, Xiang Wang, Wenmei Wang, Ning Duan

**Affiliations:** a Dentist, Department of Oral Mucosal Diseases, Nanjing Stomatological Hospital, Affiliated Hospital of Medical School, Research Institute of Stomatology, Nanjing University, Nanjing, China. Collected, analysed and interpreted the data, drafted the article and revised it critically for important intellectual content, read and approved the final manuscript.; b MMSc Candidate, Department of Oral Mucosal Diseases, Nanjing Stomatological Hospital, Affiliated Hospital of Medical School, Research Institute of Stomatology, Nanjing University, Nanjing, China. Analysed the data and revised it critically for important intellectual content, read, revised and approved the final manuscript.; c MMSc Candidate, Department of Oral Mucosal Diseases, Nanjing Stomatological Hospital, Affiliated Hospital of Medical School, Research Institute of Stomatology, Nanjing University, Nanjing, China. Analysed the data, read, revised and approved the final manuscript.; d Professor, Department of Oral Mucosal Diseases, Nanjing Stomatological Hospital, Affiliated Hospital of Medical School, Research Institute of Stomatology, Nanjing University, Nanjing, China. Provided administrative, technical and material support, read, revised and approved the final manuscript.; e Professor, Department of Oral Mucosal Diseases, Nanjing Stomatological Hospital, Affiliated Hospital of Medical School, Research Institute of Stomatology, Nanjing University, Nanjing, China. Involved in study conception and design, provided administrative, technical and material support, read, revised and approved the final manuscript.; f Associate Professor, Department of Oral Mucosal Diseases, Nanjing Stomatological Hospital, Affiliated Hospital of Medical School, Research Institute of Stomatology, Nanjing University, Nanjing, China. Involved in study conception and design, acquisition of data, analysis and interpretation of data, drafted the manuscript, read and approved the final manuscript.; †Q. Liu and H. Liu contributed equally to this work.

**Keywords:** clinical features, demographic characteristics, histopathology, oral lichen planus

## Abstract

**Purpose:**

To study the clinical and pathological characteristics of oral lichen planus (OLP) in a large sample.

**Materials and Methods:**

A comprehensive analysis was conducted on 105 patients with oral lichen planus (OLP), considering various factors including sex, age, disease site, lesion type, lesion area, morphological characteristics, self-reported symptoms, and history of systemic diseases. Histopathological examination was performed for each patient, and the pathology results were analysed according to sex and age group.

**Results:**

70.5% of the OLP patients were female, and OLP was most likely to occur in the cheek, followed by the tongue, lips, gums and palate. The patients with moderate pain according to the VAS score accounted for 60%. Thirty-nine percent of the OLP patients had a systemic disease, and the most common clinical type of OLP was nonerosive. Most of the pathological results showed liquefaction degeneration of basal cells and infiltration of lamina propria lymphocytes. There was no statistically significant difference in pathological manifestations between male and female patients, and there were statistically significant differences in pathological manifestations among different ages patients.

**Conclusion:**

This study analysed the sociodemographic data and clinical manifestations of 105 OLP patients to guide follow-up treatment planning and disease monitoring. Moreover, pathological manifestations should be analysed to avoid delayed treatment and to monitor for carcinogenesis. Furthermore, the correlation of pathological manifestations among OLP patients with different sexes and ages is conducive to further research on the specific differential manifestations and possible underlying mechanisms involved.

Oral lichen planus (OLP) is a chronic inflammatory disease of the oral mucosa ^20^^,^^25^ with a prevalence rate of 0.1%~4.0%, and is mainly found in middle-aged women.^8^^,^^15^ Most patients experience pain, discomfort from tissue roughening and other symptoms, and this pain can be aggravated by eating irritating food, which affects the quality of life. Due to the risk of cancer in some cases, it is currently considered an unequivocally potentially malignant oral disorder (OPMD) that can seriously affect the physical and mental health of patients.^6^^,^^12^^,^^17^^,^^21^ Therefore, early and accurate diagnosis of OLP is particularly important and is helpful for its treatment and prognosis. By analysing the clinical and pathological characteristics of OLP patients with a definite diagnosis, this study is expected to improve the awareness of clinicians and the diagnostic accuracy of OLP to regulate medication use early on, to reduce the risk of malignant transformation and improve the prognosis.

## MATERIALS AND METHODS

### Patients

This study included 105 patients with OLP who were admitted to the Department of Oral Mucosal Diseases (Nanjing Stomatological Hospital, Affiliated Hospital of Medical School, Nanjing University, Nanjing, China) from August 1, 2017 to December 15, 2021, after definite clinical diagnosis and pathological examination. The patients ranged in age from 28–83 years, the disease course ranged from 2~18 years, and 8 patients (7.6%) were smokers.

This investigation was approved by the Ethics Committee of Nanjing Stomatological Hospital, Affiliated Hospital of Medical School, Nanjing University (Approval No. 2016NL-047 [KS]). All patients in this study provided signed informed consent.

### Inclusion and Exclusion Criteria

The inclusion criteria for patients were as follows: (1) at least 18 years old; (2) diagnosed with OLP based on medical history, clinical manifestations and pathological examination with a VAS score >1; (3) complete clinical data available; and (4) consent to a biopsy. The exclusion criteria were: (1) other identified oral mucosal diseases or oral lichen planus classified as reticular-asymptomatic or oral lichen planus with severe abnormal hyperplasia or cancer; (2) serious systemic diseases and tumours; (3) certain drugs or silver-amalgam fillings that may cause lichenoid reactions; (4) addiction to tobacco and alcohol; and (5) any mental illness.

### Methods

The lesion location, type, area, morphological characteristics (with or without congestion erosion) and self-perceived symptoms (VAS score) of the OLP patients were collected, recorded, summarised and analysed. The diagnostic criterion was the latest OLP diagnostic criterion proposed by the American Society for Oral and Maxillofacial Pathology in 2016.^6^ A diagnosis of OLP requires the fulfilment of both clinical and histopathological criteria: clinical criteria: presence of bilateral, mostly symmetrical lesions, presence of lace-like network of slightly raised grey-white lines (reticular pattern); histopathological criteria: erosive, atrophic, bullous and plaque type lesions. Our105 patients fulfilled all these criteria. At the same time, the histopathological sections were reviewed by two pathologists and statistically evaluated.

### Statistical Methods

Excel software (Microsoft; Redmond, WA, USA) was used to sort and analyse the data. The data are expressed in %, and SPSS 26.0 software (IBM; Armonk, NY, USA) was used for statistical analysis. The chi-squared test was used to evaluate the differences in the main clinicopathological features of OLP patients according to sex and age. A p-value < 0.05 was considered to indicate statistical significance.

## RESULTS

### Study Population

The present study included 31 males and 74 females (male: female ratio 1:2.4), with ages ranging from 28 to 83 years. There were 7 patients (6.7%) aged 18–35 years, 72 patients (68.6%) aged 36–59 years, and 26 patients (24.8%) aged ≥60 years. Height was 151-188 cm, weight was 42 kg–80 kg. Among the participants, 66 rarely ate spicy foods, 7 often ate spicy foods, and 32 occasionally did so. There were 24 patients with good sleep patterns, 66 patients with normal and 15 patients with poor sleep patterns as defined by the Self-Rating Scale of Sleep (SRSS; good sleep: 22 points and below; normal sleep: 23–39 points; poor sleep: 40–50 points). Twenty patients were addicted to alcohol and tobacco. There were 3 patients with a family history of OLP. There were 41 patients with systemic diseases (hypertension/diabetes/heart disease, etc). Table 1 presents the demographic characteristics of the enrolled patients.

**Table 1:** Main demographics of patients, N (%)

Sex	
Male	31 (29.5%)
Female	74 (70.5%)
Age (years)	
18–35	7 (6.7%)
36–59	72 (68.6%)
≥60	26 (24.8%)
Sleep quality	
Good	24 (22.9%)
Normal	66 (62.9%)
Poor	15 (14.3%)
History of systemic diseases	41 (39%)

### Clinical Features

**Fig 1:**
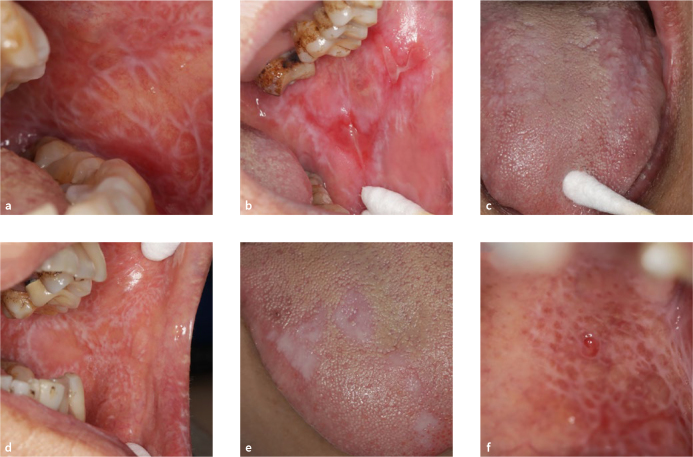
Fig 1 Clinical patterns of OLP. (a) reticular; (b) erosive/ulcerative; (c) atrophic; (d) papules; (e) plaque-like; (f) blister.

The disease course of the 105 OLP patients ranged from 2 to 18 years, of which 81 (77.1%) had the disease for less than one year after the patient started treatment, and 24 (22.9%) had the disease more than one year before starting treatement. The disease sites were the upper or lower lip mucosa (28 patients), right buccal mucosa (94 patients), left buccal mucosa (96 patients), dorsal surface of the tongue (31 patients), ventral surface of the tongue (36 patients), floor of the mouth (8 patients), hard-palate mucosa (3 patients), soft-palate or palatopharyngeal arch (10 patients), maxillary gingiva (26 patients), and mandibular gingiva (42 patients). There were 85 hyperaemic patients. Of these, 24 had hyperaemia at 1 site, 33 had hyperaemia at 2 sites, 7 had 3 sites affected, 12 had 4 sites, 2 had 5 sites, 5 had 6 sites, and 2 had hyperaemia at 8 sites. The most common clinical type was nonerosive (56 patients), and 49 patients had erosive-type lesions. Of the latter, 25 patients had 1 erosive lesion, 17 had 2, 2 had 3, 4 patients had 4, and 1 patient had 5 erosive lesions. The lesion types were noncongestive and nonerosive (19 patients), congestive or erosive (37 patients), and congestive with erosion (49 patients). The damaged area varied from 9 to 7000 mm^2^, with 71 of the patients having a damaged area ≤1050 mm^2^. The visual analogue scale (VAS) was used to measure the degree of pain associated with oral lesions: 0 = no pain, 10 = greatest pain. The higher the number is, the more severe is the pain. Self-perceived symptoms (VAS score) and mild pain (1–3 points) were reported in 19 patients. There were 63 patients with moderate pain (4-6 points) and 23 patients with severe pain (7–10 points). The specific clinical manifestations are shown in Table 2, and the typical oral manifestations of OLP patients are shown in Fig 1.

**Table 2:** Clinical manifestations

Part affected	N
Upper or lower lip	28
Right buccal mucosa	94
Left buccal mucosa	96
Dorsal surface of the tongue	31
Ventral surface of the tongue	36
Mouth floor	8
Hard palate	3
Soft palate/palatopharyngeal arch	10´
Maxillary gingiva	26
Mandibular gingiva	42
Lesion characteristics	
Hyperaemia	85 (n)
Erosive type	49 (n)
Damaged area	9–7000 (mm^2^)
VAS score (points)	N
Mild pain (1–3)	19
Moderate pain (4–6)	63
Severe pain (7–10)	23

### Histopathological Examination

**Fig 2:**
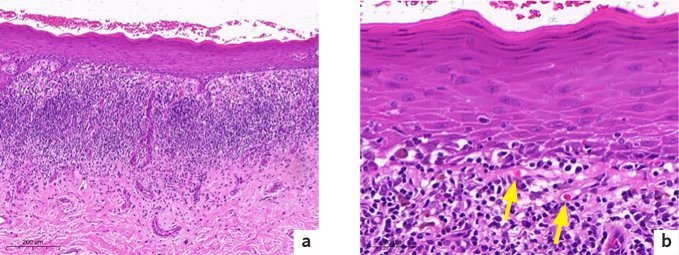
Fig 2 Histopathology of OLP. (a) Low-magnification photomicrograph showing epithelial parakeratosis and chronic inflammatory cell infiltration in the lamina propria (scale: 200 µm). (b) High-magnification photomicrograph showing liquefaction degeneration of basal keratinocytes and several colloid bodies (arrows) (scale: 50 µm).

Of the 105 patients, 44.8% had a history of treatment before biopsy, and the biopsy site was mostly buccal (88.6%). The histopathological findings of the 105 OLP patients were roughly similar and included 57 cases of chronic inflammation of the mucosal tissue, 39 cases of hyperkeratosis squamous epithelium, 43 cases of colloid bodies, and 6 cases of mild dysplasia of the squamous epithelium. There were 98 cases of liquefaction degeneration of basal cells, 8 cases of obvious granulosa layer, 8 cases of acanthosis or atrophy of the acanthosa layer, 9 cases of intraepithelial or subepithelial fissure formation, 97 cases of lamina propria lymphocyte infiltration, 2 cases of keratotic plug, 1 case of small vessel hyperplasia, 1 case of lamina propria scattered pigmentation, 1 case of focal collagen-fiber basophilic degeneration, and 1 case of partial epithelial exfoliation of the granulosa layer. The main pathological manifestations of OLP were analysed according to sex and age, and the results showed that there was no statistically significant difference in the main pathological manifestations of OLP between male and female patients. However, there were differences between the different age groups, as shown in Table 3. Figure 2 shows the typical pathological manifestations.

**Table 3:** Table 3 Main pathological manifestations of OLP in patients of different sex and age groups

Main pathological manifestations (n)	Sex		Age			Aggregate
Male (31)	Female (74)	18–35 (7)	36–59 (72)	≥60 (26)
Chronic inflammation of mucosal tissue (57)	17 (4.6%)	40 (10.8%)	3 (0.8%)	45 (13.5%)	9 (17.1%)	371
Hyperparakeratosis squamous epithelium (39)	14 (3.8%)	25 (6.7%)	2 (0.5%)	22 (6.6%)	15 (4.5%)	
Colloid bodies (43)	19 (5.1%)	24 (6.5%)	2 (0.5%)	20 (6%)	21 (6.3%)	
Liquefaction degeneration of basal cells (98 cases)	26 (7.0%)	72 (19.4%)	4 (1.1%)	70 (21%)	24 (7.2%)	
Lamina propria lymphocyte infiltration (97)	26 (7.0%)	71 (19.1%)	3 (0.8%)	70 (21%)	24 (7.2%)	
Mild dysplasia of squamous epithelium (6)	3 (0.8%)	3 (0.8%)	1 (0.3%)	3 (0.8%)	2 (0.5%)	
Obvious granulosa layer (8)	2 (0.5%)	6 (1.6%)	2 (0.5%)	4 (1.1%)	2 (0.5%)	
Acanthosis or atrophy of acanthosa layer (8)	3 (0.8%)	5 (1.3%)	1(0.3%)	4 (1.1%)	3 (0.8%)	
Intraepithelial or subepithelial fissure formation (9)	4 (1.1%)	5 (1.3%)	2 (0.5%)	4 (1.1%)	3 (0.8%)	
Keratotic plug (2)	0 (0%)	2 (0.5%)	0 (0%)	1 (0.3%)	1 (0.3%)	
Small–vessel hyperplasia (1)	1 (0.3%)	0 (0%)	1 (0.3%)	0 (0%)	0 (0%)	
Lamina propria scattered pigmentation (1)	0 (0%)	1 (0.3%)	0 (0%)	1 (0.3%)	0 (0%)	
Focal collagen–fiber basophilic degeneration (1)	0 (0%)	1 (0.3%)	0 (0%)	1 (0.3%)	0 (0%)	
Partial epithelial exfoliation of granulosa layer (1)	1 (0.3%)	0 (0%)	0 (0%)	0 (0%)	1 (0.3%)	
χ^2^ value	13.920		53.287			
p–value	0.380		0.001*			

*p < 0.05 indicates statistical significance.

## DISCUSSION

OLP is a chronic inflammatory mucosal disease with an unknown aetiology. Most previous studies have shown that OLP tends to occur in middle-aged people and is more common in women.^5^^,^^14^^,^^18^ Among the 105 patients included in this study, OLP was found in 68.6% of the people aged 36-59 years. There were more women than men, and the male:female ratio was 1:2.4, which is basically consistent with the findings of recent reports. This may be because the onset of OLP is associated with changes in oestrogen levels in women.^20^

Genetic background may play a role in OLP pathogenesis, as several familial cases have been reported;^24^ however, the association has not been consistent. Researchers have found that some HLA antigen sites in OLP patients are abnormal, and the frequency of HLP-B5 and HLA-B8 sites is increased. Genetic polymorphisms of several cytokines have been postulated to be associated with the clinical presentation of OLP.^23^ It has been reported that genetic polymorphisms of the first intron of the promoter gene of interferon-gamma (IFN-γ ) may be an important risk factor for developing OLP,^3^^,^^4^^,^^13^ whereas an increase in the frequency of the 308A tumour necrosis factor-alpha (TNF-α) allele may contribute to the development of additional skin involvement.^4^ In recent years, it has been reported that OLP is closely related to HLA-DR1. This study revealed that patients with a family history of lichen planus accounted for 2.9% of the total population, suggesting that there may be genetic factors involved in lichen planus pathogenesis, and further analysis should be conducted from the perspective of genetic information.

Several reports suggest that OLP patients are more likely to develop diabetes than healthy people are, and some scholars also suggested that lichen planus is related to diabetes.^2^^,^^27^ In this study, the incidence of hyperglycaemia and diabetes in OLP patients was 1.9%, which was lower than the incidence of diabetes in China (more than 9.7%), possibly due to the small sample size. Some scholars^15^ have proposed that the onset of OLP is related to *Helicobacter pylori* infection, reporting that the type of OLP damage is related to the severity of gastritis and that oral damage can improve after chronic gastritis treatment. This study revealed that gastrointestinal diseases (including various types of gastritis and gastrointestinal ulcers) are closely related to OLP in clinical practice and can be further studied.

Other studies have shown that sex, age, thyroid disease, and hypertension are related to OLP. In this study, 7.6% of the OLP patients had hypertension, and 2.9% of the patients had thyroid-related diseases. These correlations should be further studied in the future.

OLP is a multiform and multisite disease. According to the REU (reticular, erosive, ulcerative) score, the oral mucosa is divided into 10 parts: upper/lower lip mucosa, maxillary gingiva, mandibular gingiva, left buccal mucosa, right buccal mucosa, dorsal surface of the tongue, ventral surface of the tongue, floor of the mouth, hard-palate mucosa and soft palate/lingual tonsil. In this study, OLP was most likely to occur in the cheek, followed by the tongue, lips, gums and palate. Xue et al^29^ analysed OLP medical records from 1963 to 2003, and the conclusions were basically consistent with the results of this study. Studies have shown that 86.4% of OLP patients have involvement of the buccal mucosa, 31.6% have involvement of the tongue, and fewer patients have involvement of only the gums.^26^ Eisen^8^ suggested that the order of OLP occurrence was buccal, tongue, gum and lip, possibly because of differences in geography, race and sample size.

OLP is an underlying malignant disease of the oral mucosa (OPMD), so early diagnosis is particularly important for the treatment and prognosis of OLP patients. Clinically, there are six clinical subtypes of OLP that can be observed individually or in combination: reticular, plaque-like, atrophic, erosive/ulcerative, papular and bullous (Fig 1).^1^ The most common of these subtypes are the reticular, erosive/ulcerative and plaque-like subtypes.^11^^,^^22^ The reticular lesions, the most recognised form of OLP, are often asymptomatic and appear as multiple papules with a network of small, raised, whitish-grey, lacy lesions referred to as Wickham striae.^10^ In the absence of the classic reticular pattern on oral mucosal surfaces, it is challenging to clinically diagnose OLP.^22^ Histological confirmation of the diagnosis is thus needed. The erosive form of OLP may present with erythema caused by inflammation or epithelial thinning, and ulceration/pseudomembrane formation can also be observed, with the periphery of the lesion surrounded by reticular keratotic striae.^26^ Atrophic and erosive/ulcerative OLP lesions result in varying degrees of discomfort. The plaque form of OLP mimics leukoplakia in that it appears as a white, homogeneous, slightly elevated, multifocal, smooth lesion. The plaque form of OLP commonly affects the tongue and buccal mucosa.^26^ These particular OLP lesions rarely remit spontaneously and may lead to confusion with other vesiculo-bullous diseases (such as paraneoplastic pemphigus, etc) that share similar clinical features.^9^ When the clinical manifestations of OLP are atypical or when multisite lesions occur and the clinical manifestations are not very similar, misdiagnoses and missed diagnoses can easily occur. Therefore, most scholars suggest that the diagnosis of OLP should be combined with clinical and histopathological examination to help identify other diseases and eliminate the risk of malignant transformation.^22^

At present, there are three sets of pathological diagnostic criteria for OLP used in clinical practice. The histological diagnostic criteria for oral lichen planus were first proposed by the World Health Organization in 1978; these criteria emphasise ribbon-like infiltration dominated by lymphocytes and liquefaction degeneration of basal cells. However, van der Meij et al^28^ reported that this diagnostic standard was highly subjective and insufficient in terms of repeatability. In 2003, van der Meij et al^19^ proposed a modified oral lichen planus standard in which “no epithelial dysplasia” was added to the original standard.^28^ At present, this standard is widely used outside China countries and recommended in China. In 2016, the American Society of Oral and Maxillofacial Pathology proposed new diagnostic criteria for oral lichen planus based on the first two criteria.^7^ The new diagnostic criteria still focus on band infiltration of lymphocytes and liquefaction degeneration of basal cells as the basic features of the diagnosis of oral lichen planus, while emphasising that “epithelial wart growth” and “abnormal epithelial hyperplasia” should be excluded from the diagnostic criteria.

Based on the 2016 pathological diagnostic criteria for OLP, this study analysed the demographic and sociological data and clinical manifestations of 105 patients diagnosed clinically and pathologically with OLP in our hospital from 2017 to 2021. This approach is beneficial for formulating follow-up treatment plans and monitoring patient conditions. According to the results of this study and related studies from outside China, regardless of whether the clinical manifestations of OLP are typical, a biopsy should be performed to distinguish OLP from other diseases if the conditions of the first diagnosis permit and to provide a basis for accurate clinical treatment and follow-up cycles.

At present, there are no relevant studies on the pathological manifestations of OLP patients according to age or sex. Based on the statistical analysis of the pathological manifestations of OLP, this study included two factors, sex and age. We found no statistically significant differences in the major pathological manifestations between male and female OLP patients; moreover, there were differences in the major pathological manifestations of OLP patients at different ages, and the specific differences need to be further studied.

This study has several limitations, such as a small sample size, and it is still impossible to reach a definitive conclusion. On this basis, different pathological manifestations should be studied further.

## CONCLUSION

Correlation analysis of the pathological manifestations of OLP patients of different sexes and ages revealed no statistically significant difference in the main pathological manifestations of OLP in male and female patients, but there were differences in different age groups. The specific differential manifestations and possible underlying mechanisms will be further studied in the future.
